# Macular Integrity Assessment and Fixation Analysis in Chronic Central Serous Chorioretinopathy

**DOI:** 10.1155/2018/9479848

**Published:** 2018-03-14

**Authors:** Joanna Dolar-Szczasny, Anna Święch-Zubilewicz, Jerzy Mackiewicz

**Affiliations:** Department of Retina and Vitreous Surgery, Medical University of Lublin, Lublin, Poland

## Abstract

**Purpose:**

To investigate retinal sensitivity characteristics associated with morphologic changes in the eyes exhibiting chronic central serous chorioretinopathy (CSC), using macular integrity assessment (MAIA) microperimetry.

**Methods:**

A retrospective, cross-sectional, observational study was constructed. The eyes of patients classified as chronic CSC, according to the onset of subjective symptoms with serous retinal detachment, as confirmed by optical coherence tomography examination, were included in the study. Retinal sensitivity and fixation were analyzed by performing microperimetry examinations using the MAIA instrument.

**Results:**

We reviewed microperimetry examinations of 15 eyes of 15 patients (age: 28–51 years) with chronic CSC and mean best-corrected visual acuity of −0.2 logMAR units. The mean retinal integrity in the chronic CSC group was 49.0 ± 27.6, which was significantly different from the control eyes. The mean average threshold in the eyes with chronic CSC was 24.7 ± 5.8 dB, which also was significantly different from the control eyes. Fixation stability was significantly different between the CSC and control eyes for the P1 parameter (90.1 ± 13.7 versus 99.3 ± 1.5), and for the P2 parameter (97.4 ± 4.0 versus 100.0 ± 0.0).

**Conclusion:**

New microperimetry technology may provide valuable information regarding the visual status of chronic CSC cases. Our findings suggest that retinal sensitivity and fixation stability in chronic CSC eyes may serve as useful indicators for assessing the effectiveness of clinical treatments.

## 1. Introduction

Central serous chorioretinopathy (CSC) is a macular disease that typically affects young and middle-aged adults. It is characterized by a serous retinal detachment at the posterior pole. Patients complain of blurred vision in the central or paracentral visual field and varying degrees of metamorphopsia, micropsia, central scotoma, and low-contrast sensitivity. In the majority of patients, CSC is self-limiting, and visual acuity recovers fully after subretinal fluid reabsorption [1]. However, in some patients, serous retinal detachment does not resolve for many months; even those with good visual acuity can experience a reduction in other visual functions, for example, contrast sensitivity, color discrimination, dark adaptation, focal macular electroretinograms, and macular sensitivity. In a small percentage of patients with CSC, prolonged recovery and recurrent episodes may lead to severe visual loss [2–8].

CSC is likely caused by choroidal vascular hyperpermeability that can be visualized on fluorescein angiography (FA) by focal leakage at the level of the retinal pigment epithelium (RPE) [9]. Some patients may present with multiple pinpoint leaks in FA or with a smokestack fluorescein pattern. FA may also show evidence of previous CSC episodes limited to the extramacular area; these may go undetected, as they are asymptomatic. In patients with long-standing CSC, findings may include focal or diffuse RPE atrophy and areas of RPE pigment clumping. Other notable findings include evidence of gravity-driven descending tracts of subretinal fluid on FA or fundus autofluorescence (FAF) images. These tracts are initially hyperautofluorescent in an acute phase of the disease, then become increasingly hypoautofluorescent as RPE cells are damaged within the area of fluid leakage. The staining of the inner choroid within the macular region and in the periphery, as seen on mid-phase indocyanine green (ICG) angiography, is the primary evidence of choroidal hyperpermeability. Focal or diffuse RPE defects in cases of chronic CSC are best visualized in FAF images, which show hypofluorescence spots in the damaged area. Subretinal fluid presence, due to neurosensory retinal detachment, can be visualized and monitored by noninvasive optical coherence tomography (OCT) [10]. Enhanced depth imaging spectral-domain optical coherence tomography (SD-OCT) findings demonstrate a very thick choroid in both eyes of patients with CSC [11]. The acute phase of CSC demonstrates a self-limiting, natural outcome; in contrast, the chronic form of CSC—with sustained subretinal fluid—may cause permanent visual disturbances. Therefore, analyzing functional parameters of the chronic form of CSC is important for both timing of treatment and prediction of prognosis.

Typically, CSC is classified as chronic through subjective patient history, and there has not yet been a definite, objective method to estimate the extent of subretinal fluid that might cause retinal dysfunction. Microperimetry is a very advanced technique for evaluating retinal sensitivity. Currently, it is considered a useful tool in many ophthalmologic clinical trials; notably, 78 clinical trials, in the clinical trial registry of the United States National Institutes of Health, use microperimetry evaluation—five of these involve CSC. A better understanding of the chronicity of CSC is important in determining treatment options; thus, we analyzed the retinal sensitivity of patients with chronic CSC, according to the onset of subjective symptoms, using macular integrity assessment (MAIA) microperimetry.

MAIA is a third-generation fundus perimeter, which permits differentiation between normal age-related loss of macular sensitivity and pathological (disease state) changes that require treatment. It provides a first step in retinal disease management, thereby enabling clinicians to test macular function and follow sequential changes in retinal disease progression. This will improve diagnosis and treatment of patients by permitting the detection of disease changes at an earlier stage than can be achieved using conventional testing methods. In this study, we aimed to present retinal sensitivity characteristics associated with morphological changes in CSC, using MAIA microperimetry.

## 2. Material and Methods

We retrospectively reviewed medical records of patients with idiopathic CSC, who were examined at the Retinal and Vitreous Surgery Department of Medical University in Lublin, from December 2014 to June 2017. Idiopathic CSC was diagnosed based on the following: presence of a serous detachment of the neurosensory retina involving the macula, as demonstrated by SD-OCT; leakage at the level of RPE on FA; and indirect ophthalmoscopy. We analyzed the examinations of patients who were observed for >6 months where subretinal fluid was still present. Only the eyes that presented with subretinal fluid in the foveal area, on OCT, were included in this study. Exclusion criteria were corneal or lens opacities, glaucoma or ocular hypertension, and a history of posterior uveitis, retinal detachment, ocular trauma, or optic neuropathy. None of the patients had been previously treated by laser photocoagulation or photodynamic therapy.

**Figure 1:**
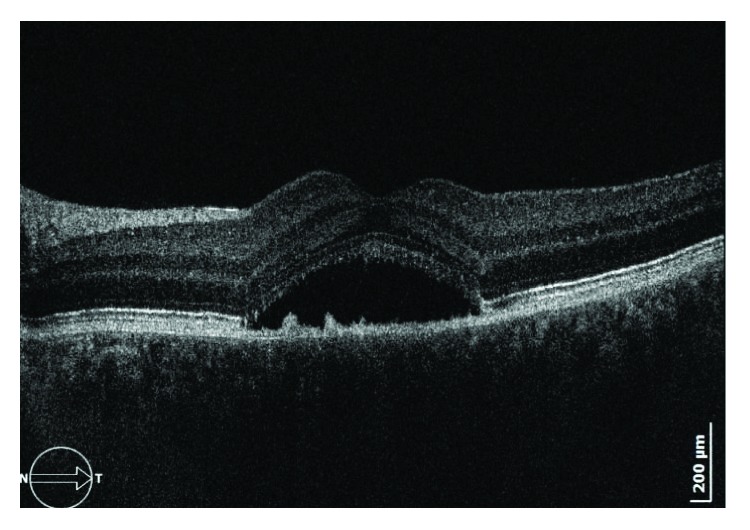
Optical coherence tomography of the macular region with subretinal fluid.

**Figure 2:**
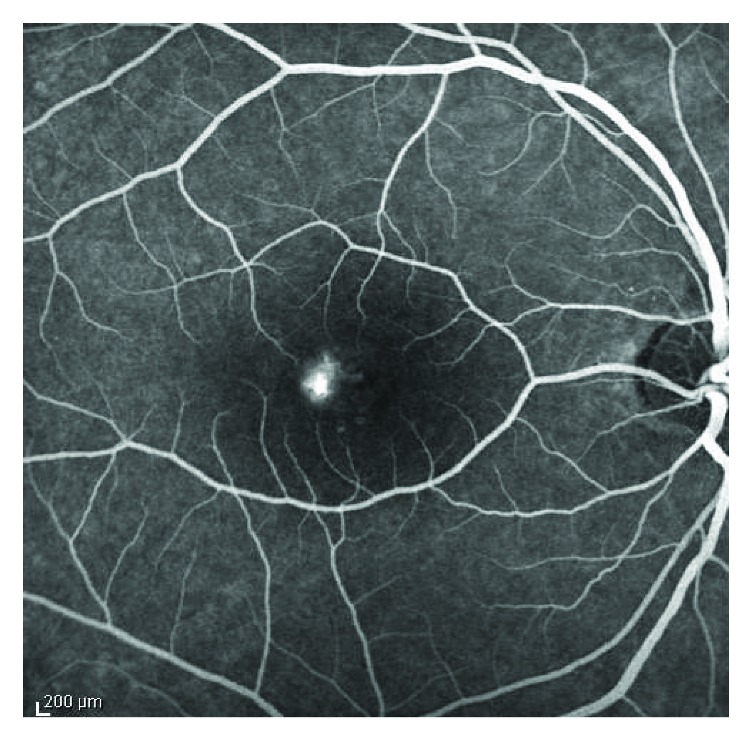
Fluorescein angiography of the eye with central serous chorioretinopathy.

SD-OCT was performed using an SOCT Copernicus HR (OPTOPOL Technology, Poland) cube volume scan (Figure 1). FA and FAF images were obtained using an HRA2 confocal scanning laser ophthalmoscope (Heidelberg Retina Angiograph, Heidelberg, Germany) (Figure 2).

In addition to undergoing a comprehensive ophthalmologic examination—including best-corrected visual acuity (BCVA) measurement with EDTRS charts, slit lamp biomicroscopy, and indirect ophthalmoscopy—all patients had undergone microperimetry examination.

**Figure 3:**
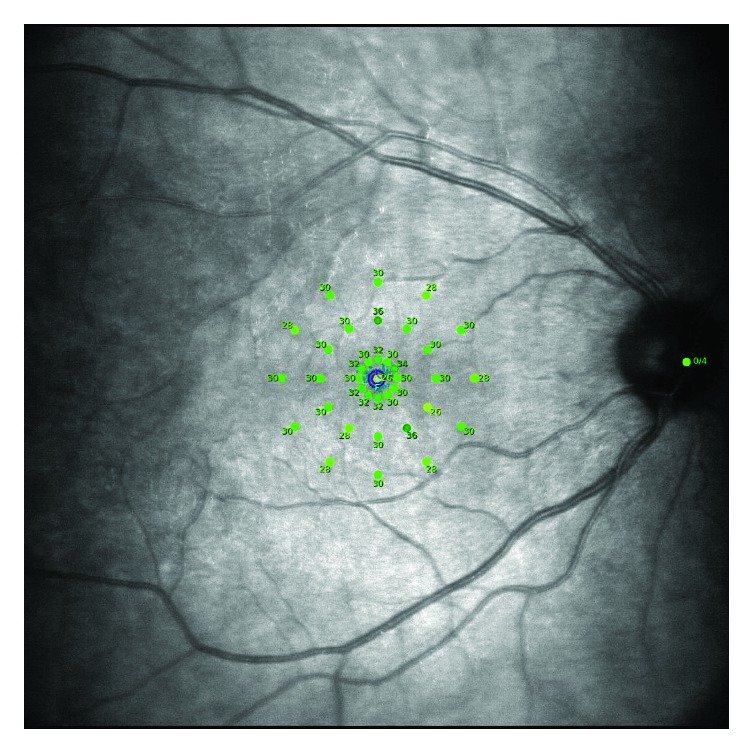
Microperimetry examination of the eye with central serous chorioretinopathy performed with the macular integrity assessment instrument.

Microperimetry examinations were performed using the MAIA (CenterVue, Padova, Italy) microperimeter (Figure 3).

All examinations and data collection were performed in accordance with the 2000 revision of the Declaration of Helsinki. Approval was obtained from the Institutional Review Board of Medical University in Lublin, Poland.

### 2.1. Microperimetry Examinations

Microperimetry testing was performed using standardized mesopic testing conditions. Patients were instructed about the procedure, and tests were conducted with the examiner viewing the fundus on the device monitor in real time, while the patient was shown test stimuli. The examination was performed in a darkened and quiet room, following pupil dilatation.

The following parameters were used in the test: a 37-stimulus grid overlying the central 10°, Goldmann III stimulus with a duration of 200 ms, 4-2 threshold strategy, and a 1° diameter red-circle fixation target. The standardized stimulus grid was composed of a single central foveal response and three concentric rings of retinal loci at 2°, 6°, and 10° from the center. Average sensitivity values for the macular region were calculated on the basis of the total projected stimulus mean value (0–10° from the fixation point) and are presented as an average threshold value in decibels (dB). MAIA 4-2 follows the perimetric standard: it changes light intensity in 4 dB steps until there is a change from not seen to seen stimuli (or from seen to not seen). Then, it changes the intensity in 2 dB steps until the stimulus is not seen again (or seen again). The standard MAIA examination, using the 4-2 strategy, has an average duration of 5.5 minutes.

A unique parameter of the MAIA microperimeter is the macular integrity index, which is a proprietary statistical value that is calculated by use of a neural network multivariate model (the EYEdBTM). The model includes age, average threshold value, a measurement of points with threshold < 25 dB, and all measured threshold values; it is derived by comparison with the manufacturer's normative data and describes the likelihood that threshold values will differ significantly from normal values. The algorithm of the macular integrity index calculation is not published. The macular integrity index is a numerical value that describes the likelihood that a patient's responses are normal, suspect, or abnormal, when compared with age-adjusted normative data. MAIA automatically generates a macular integrity index, which is calculated using the number of stimuli lower than 25 dB, the sensitivity of the central stimuli, and the fixation stability factor. Alone, the macular integrity index does not represent the severity of the disease process. Higher numbers suggest a greater likelihood of pathological findings, while lower values suggest a greater likelihood of normal findings. Macular integrity index reflects the functional status of the eye with macular disorder, revealing morphological alterations in the macular region. There is no direct relationship between the average threshold value and the macular integrity index. In fact, it is possible for the average threshold value to be normal in a test subject who exhibits an abnormal macular integrity index. Notably, the macular integrity index is only present in examinations that are performed using the standard MAIA stimuli grid and the 4-2 projection strategy.

Fixation stability was assessed by tracking eye movements 25 times/sec and plotting the resulting distribution over the scanning laser ophthalmoscope image. Fixation characteristics were calculated automatically by the MAIA microperimetry software, after a landmark had been located in the center of the fovea. Fixation stability (P1 and P2) was measured by calculating the percentage of fixation points (%) located within a distance of 1° and 2°, respectively. Automatic classification of stability was based on the following criteria: (1) if >75% of the fixation points were located within P1, fixation was classified as “stable”; (2) if <75% of the fixation points were located within P1, but >75% of the fixation points were located within P2, fixation was classified as “relatively unstable”; and (3) if <75% of the fixation points were located within P2, the fixation was classified as “unstable” [12].

### 2.2. Data Analyses

Data are presented as mean values with standard deviation (±SD). Results from age-matched control eyes (age range, 24–47 years; mean age, 39 years) and eyes with chronic CSC were compared by Student's *t* test.

All statistical analyses were performed using STATISTICA 12 statistical software (StatSoft Polska, Krakow, Poland). Statistical significance was defined as a *p* value < 0.05.

## 3. Results

A total of 15 eyes of 15 patients with chronic CSC were included in this study. The mean age of the patients in the study was 40.6 years (range, 28–51 years; median age, 41 years). A total of 13 patients (86.7%) were men, and two patients (13.3%) were women. Ten of 15 patients exhibited focal hypofluorescence changes within FAF images. Because of poor fixation stability in patient number 1, we excluded the patient's data from our statistical analysis. Detailed clinical profiles of chronic CSC patients and control subjects are presented in Tables 1 and 2.

In the eyes of CSC patients, the mean BCVA at the time of microperimetry examinations was 0.2 logMAR units (range: 0.7–0.0; median: − 0.2 ± 0.2), with a statistically significant difference between CSC eyes and control eyes. The mean retinal integrity index in the eyes of CSC patients was 49.0 ± 27.6, which was statistically different from the control eyes (2.6 ± 1.6) (*p* < 0.05). The mean average threshold in the eyes of CSC patients was 24.7 ± 5.8 dB, which was statistically significantly lower than that in the control eyes (31.4 ± 2.7 dB) (*p* < 0.05). Fixation stability in most cases was classified as stable but was statistically different between P1 (CSC eyes: 90.1 ± 13.7 versus control eyes: 99.3 ± 1.5) and P2 (CSC eyes: 97.4 ± 4.0 versus control eyes: 100.0 ± 0.0) parameters (Table 3).

## 4. Discussion

When evaluating patients with macular diseases, measurement of retinal sensitivity and fixation stability by microperimetry is likely to provide a more precise examination than determination of simple visual acuity [13], as visual acuity is measured using high-contrast optotypes under bright light conditions, and, therefore, does not fully represent the visual functions of patients in their daily lives.

The MAIA microperimeter is a relatively new instrument that couples digital fundus imaging with automated microperimetry. To our knowledge, this study is the first to report both examination of patients with chronic CSC and analysis of the macular integrity index, using the MAIA microperimeter. Another study that used MAIA to examine patients with acute CSC reported that microperimetry of <20 dB had a relative risk of 4.5 for development of subretinal fluid persistence [14]. All microperimetry tests were performed with dilated pupils, as pupil dilatation does not affect the test results.

CSC is regarded as a self-limiting disease, with good prognosis after subretinal fluid resolution. However, in chronic cases, persistent serous detachment of the macular region may cause RPE damage and permanent visual dysfunction [15]. Recent microperimetry studies have shown that eyes with resolved CSC may exhibit significantly lower central retinal sensitivity, even after achievement of good central visual acuity [16, 17]. In contrast, we analyzed patients who exhibited presence of chronic subretinal fluid. Our analysis shows that the eyes with chronic CSC can exhibit lower macular sensitivity values in the central macula, compared with the control eyes; this is consistent with observations of decreased VA. There was also statistical evidence that the index of macular integrity was different between the CSC eyes and control eyes; however, this measurement does not reflect the severity of CSC pathology and has no correlation with average threshold values. Finally, fixation parameters were worse not only for the central point of fixation (P1) in the chronic CSC eyes. P2 parameters were also outside normal limits, indicating that fixation stability in central and paracentral area was impaired.

Our study has several limitations, including a relatively small sample size and retrospective nature. Additionally, we could not correlate reductions in retinal sensitivity with patients' subjective symptoms. However, our study is notable for reporting parameters of macular dysfunction, in cases of chronic subretinal fluid presence, as part of natural history of the disease, which contrasts with studies that have reported posttreatment microperimetry results in the chronic CSC eyes [18–22]. Critically, prospective studies, with a larger number of cases, are required to confirm the applicability of these results.

## 5. Conclusion

Our findings are important in the determination of retinal sensitivity and fixation stability in the chronic CSC eyes that have experienced extended exposure to subretinal fluid. These parameters may be useful indicators for assessing the effectiveness of clinical treatments.

## Figures and Tables

**Table 1:** Clinical characteristics of patients with chronic central serous chorioretinopathy.

Patient number	Age (years)	Visual acuity (logMAR)	Macular integrity	Average threshold (dB)	Microperimetry fixation stability P1 (%)	Microperimetry fixation stability P2 (%)
2	40	0.18	48.5	27.3	100	100
3	35	0.0	13.7	29.0	94	99
4	43	0.1	53.4	27.7	72	90
5	36	0.2	93.6	25.5	100	100
6	43	0.7	52.6	12.1	76	95
7	47	0.1	35.0	28.6	99	100
8	40	0.18	92.0	26.1	100	100
9	35	0.18	7.6	29.4	82	97
10	48	0.3	58.4	22.5	100	100
11	36	0.2	41.6	28.1	94	100
12	41	0.5	65.6	13.7	90	95
13	42	0.3	81.4	16.5	56	88
14	45	0.2	56.4	27.3	99	100
15	51	0.2	26.9	27.5	100	100

**Table 2:** Clinical characteristics of control subjects.

Patient number	Age (years)	Visual acuity (logMAR)	Macular integrity	Average threshold (dB)	Microperimetry fixation stability P1 (%)	Microperimetry fixation stability P2 (%)
1	36	0.0	3.6	30.2	99	100
2	35	0.0	0.2	35.5	100	100
3	42	0.0	1.4	30.8	100	100
4	43	0.0	2.8	28.2	100	100
5	35	0.0	1.2	29.6	100	100
6	38	0.0	5.0	32.7	100	100
7	24	0.0	3.8	31.8	98	100
8	41	0.0	4.2	27.8	100	100
9	43	0.0	2.4	33.8	97	100
10	47	0.1	1.2	29.6	100	100
11	40	0.0	5.6	34.2	95	100
12	39	0.0	2.2	35.2	100	100
13	37	0.0	1.8	32.8	100	100
14	46	0.1	0.4	31.6	100	100
15	42	0.0	3.4	26.8	100	100

**Table 3:** Mean visual acuity, macular integrity index, average threshold, and fixation stability in eyes with chronic central serous chorioretinopathy and control eyes.

	Control eyes (mean ± SD)	Eyes with chronic CSC (mean ± SD)	Statistic (*t* test)	*p* value
Visual acuity (logMAR)	0.0 ± 0.0	0.2 ± 0.2	0.00	*p* < 0.05
Macular integrity	2.6 ± 1.6	49.0 ± 27.6	0.00	*p* < 0.05
Average threshold (dB)	31.4 ± 2.7	24.7 ± 5.8	0.01	*p* < 0.05
Fixation stability P1 (%)	99.3 ± 1.5	90.1 ± 13.7	0.01	*p* < 0.05
Fixation stability P2 (%)	100.0 ± 0.0	97.4 ± 4.0	0.02	*p* < 0.05
